# Perception of Shared Governance Among Registered Nurses in Ambulatory Care Center at a Tertiary Care Hospital in Saudi Arabia

**DOI:** 10.7759/cureus.8736

**Published:** 2020-06-21

**Authors:** Bayan Kaddourah, Mohamad Al-Tannir, Shadi Kakish, Isamme AlFayyad

**Affiliations:** 1 Nursing Affairs, King Fahad Medical City, Riyadh, SAU; 2 Ambulatory Care, American University of Beirut Medical Center, Beirut, LBN; 3 Epidemiology and Public Health, King Fahad Medical City, Riyadh, SAU; 4 Research Center, King Fahad Medical City, Riyadh, SAU

**Keywords:** nursing, quality of health care, decision making, saudi arabia, power

## Abstract

Background

Shared governance is considered a model for mounting autonomous decision making in nursing profession and practice. This study aimed to assess how registered nurses in an outpatient department in a tertiary care hospital perceive shared governance.

Methods

We conducted a cross-sectional study among a convenient sample of registered nurses in an outpatient department. A self-administered, Index of Professional Nursing Governance (IPNG) questionnaire was used to measure the study outcome. A descriptive analysis was used to describe nurses' characteristics and study outcomes.

Results

A total of 186 nurses completed the questionnaire. Of whom, 151 (92.1%) were female, and 78 (47.3%) were aged between 20 and 30 years. Only 54 (29.3%) and 59 (31.7%) had indicated a shared decision in terms of controls and influence scales, respectively. The majority of the nurses indicated traditional shared across shared governance scales except in the access information scale.

Conclusion

The findings showed a prevalent traditional nursing management style in the study setting. Supportive strategies and education must be provided for both managers and staff nurses to develop and implement shared governance in their practice.

## Introduction

In a complex health care system, nurses are a vital component in providing optimal patient quality care and promote outcomes. Nursing nowadays facing multifaceted challenges, like shortages in the workforce, increased workloads, and patient acuities that have reshaped the attention to the quality of nursing care while fulfilling increased patient needs and demands. In addition to these challenges, nurses are astounded with increased rules that inflate their work profile and load, increase job dissatisfaction, and decrease bedside time spent with the patient. Although these challenges increased nurses' responsibility and accountability to accomplish professional practice, it was not escorted with increasing power or authority to figure out required changes to impact nursing practice [1].

Therefore, healthcare organizations have developed several professional practice models to direct disciplines' clinical practice, empowering and authorizing health staff, and improve provided quality of care [2,3]. The nursing profession has encouraged and supported nursing involvement in nursing practice models such as shared governance [4]. Nurse participation in shared governance guarantees accountability for the safety and quality of care and the autonomy of the nurses [4].

Shared governance has been defined as "a decentralized approach which gives nurses a greater authority and control over their practice and work environment; engenders a sense of responsibility and accountability; and allow active participation in the decision-making process, particularly in the administrative area from which they were excluded previously" [5]. Governance generally comprises structure and process where a group of individuals direct, control, and regulate their goal-oriented efforts [6]. Shared governance as an official program should include nurses in control and authority decisions by attaining the right to control their practice and extending their governance beyond that to higher and different tasks like scheduling, and evaluating personnel, budgeting, that were historically controlled solely by managers [6]. Moreover, shared governance is a model that includes shared decision-making between the healthcare workforce members and is centered on the principles of partnership, equity, accountability, and ownership [7,8].

Shared governance structure supports patient care directly, promotes nurses’ control over their practice and accountability for quality patient care [2]. For nurses, it results in feelings of empowerment that allow for professional autonomy. The application of shared governance structure leads to enhance the provision of quality of care [4,9], cultivate collaboration between healthcare professionals, advance the quality of care and clinical effectiveness; upsurge employees confidence; assist in developing individual and professional skills; growth of professional profile; which in turn lead to improve personal communication; advance knowledge and skills; intensification of professionalism and accountability; reduce duplication of effort [4,10,11].

Nurses’ satisfaction is a vital end-point of shared governance. Investigating shared governance among nurses at King Fahad Medical City is important because of the influence of attention on human factors in nursing and maintain workforce retention. Therefore, this study aimed to assess the perception of shared governance among nurses in a tertiary care hospital, Riyadh, Saudi Arabia.

## Materials and methods

Study design and settings

A cross-sectional study was conducted to assess perceptions of shared governance levels among nurses. A convenient sampling method was used for recruiting the nurses working in ambulatory care in a tertiary care hospital, Riyadh, Saudi Arabia. All ambulatory care nurses were invited to participate in the study by an invitation letter attached to a copy of the questionnaire and cover sheet describing the study’s objective and voluntary participation. Participants' identity was kept anonymous. 

Study instrument

The Index of Professional Nursing Governance (IPNG) scale was used in this study [6]. The IPNG scale includes 86-questions measuring the perceptions of shared governance on a continuum from traditional, to shared, and to self-governance. The scoring was based on a 5-point Likert scale as follows:

1 = "Nursing management/administration only"

2 = "Primarily nursing management /administration with some staff nurse input"

3 = "Equally shared by staff nurses and nursing management/ administration"

4 = "Primarily staff nurses with some nursing management/administration"

5 = "Staff nurses only"

Likert scores of "1 and 2" mean decision making controlled by management/administration. Scores higher than "3" mean more nurses involvement in decision making. IPNG scale measures six-dimensions of shared governance containing (1) “controls, (2) influences, (3) official authority, (4) participates, (5) access to information (6) ability.

The IPNG results are summed to give an overall score for shared governance as follow:

§ 86-172 indicates traditional (management) decision-making environment.

§ 173-344 indicates a setting which uses shared decision making between nurses and management

§ 345-430 indicates nurses are the decision-making group.

Data collection

All nurses were invited to participate in the study between the period of June 2017 and September 2017. An invitation letter attached to a copy of the questionnaire and cover sheet describing the study’s objective and voluntary participation. A trained research coordinator approached all eligible nurses to participate in the study. Completed questionnaires were collected in distributed boxes in all clinics.

Data analysis

Data were analyzed using SPSS 22.0 software (SPSS Inc., Chicago, IL, USA). Frequency and percentages were used for describing demographics characteristics and levels of nursing shared governance.

Ethical considerations

The institutional review board approved the study at King Fahad Medical City, and the anonymity of the participants was maintained. Participants' completion of the questionnaire implied their consent. Institutional Review Board approval was secured from King Fahad Medical City before the study was conducted.

## Results

Sample characteristics

A total of 186 nurses included in the study. The majority of participating nurses 151 (92.1%) were females, and 148 (92.8%) were staff nurses. About 78 (47.3%) of the nurses were aged between 20 and 30 years. One-hundred and thirty (79.9%) had a bachelor degree, while 25 (15.2%) had a nursing diploma. Nearly half of the nurses had six-ten years of experience, and 119 (72.5%) had one-five years of experience in the current institution (Table 1).

**Table 1:** Participants’ demographics

Demographics	n(%)
Gender	
Male	13 (7.9)
Female	151 (92.1)
Age (year)	
20-30	78 (47.6)
31-40	44 (26.8)
41-55	42 (36.6)
Level of Education	
Diploma	25 (15.2)
Bachelor	130 (79.3)
Postgraduate	9 (5.5)
Position	
Head Nurse	6 (3.7)
Charge Nurse	10 (6.1)
Staff nurse	148 (92.8)
Total years of experience (year)	
1-5	29 (16.7)
6-10	81 (49.4)
>10	54 (32.9)
Years of experience in the current institution (year)	
1-5	119 (72.5)
6-10	17 (10.4)
>10	28 (17.1)

Table 2 displays the descriptive statistics for the six scales of the IPNG scale. Regarding the “Control” scale, the results showed that slightly more than two-thirds (68.5%) of the nurses (n=126) perceived traditional decision making and 54 (29.3%) had indicated a shared decision making. Furthermore, 122 (65.6%) of the nurses perceived traditional decision making in the “Influence” scale compared to 59 (31.7%) who perceived a shared decision making.

The results of “Participation” scale indicated that more than half of the nurses (54.1%) perceived having a traditional decision making. Moreover, the “Ability” scale showed a traditional decision making among 110 (59.5%) of the nurses. Nevertheless, 72 (39.9%) of the nurses revealed the “Ability” scale is governed by the shared decision making. (Table 2)

**Table 2:** Sub-scales of nursing shared governance

Factor scales	Traditional (management) decision making (86–172) n(%)	Shared decision making (173–344) n(%)	Nurses are the decision maker (345-430) n(%)
Controls	126 (68.5)	54 (29.3)	4 (2.2)
Influence	122 (65.6)	59 (31.7)	5 (2.7)
Official authority	124 (67)	56 (30.3)	5 (2.7)
Participation	100 (54.1)	81 (43.8)	4 (2.1)
Access information	74 (40.2)	105 (57.1)	5 (2.7)
Ability	110 (59.5)	72 (39.9)	3 (1.6)

## Discussion

Overall, the study showed a traditional management decision making as indicated by the nurses. The distributions of the percentages of nurses' responses were all more or less asymmetrical and concentrated to the left side of the chart, indicating a traditional model of governance. The most proportioned distribution that makes relatively somewhat balance between nurses' shared governance model, and the traditional model of governance was for the participation scale.

Overall, the findings reflect the administrative driven model of governance at the study site, which means that the governance and management type had no effect on the perception of staff nurses and that nurses managers are working in an environment that is not equally sharing decisions and not enabling staff nurses to control over their practice.

In the control scale, the results indicated that nurses had limited control over their practice. The findings were inconsistence with the similar studies reported in the literature which revealed that nurses' perceptions of their work setting are more related to the shared governance model [12,13]. These studies indicated that nurses and administration were equally involved in decision-making activities concerning their control over professional practice [12,13].

Relating to the influence over practice and resources, participating nurses perceived they lack influence or formal authority in several daily procedures, including patient care locations, obtaining and monitoring supplies, patients’ admissions, and discharges, creating new clinical and administrative positions, and generating schedules. The results disagree with Hashish et al., who reported that nurses indicated high influence over practice and resources [12]. Along with the findings of the current study align with Seada and Etway who showed that nurses perceived various parts of their influence over different activities are being done only with an administrator decision with limited staff inputs [14].

The findings of our study showed that nurses perceived a lack of shared ability with nursing management to engage in nursing profession committees mostly concerning their clinical practices, staff scheduling, and strategic planning. Moreover, nurses perceived they have limited ability to play a part in committees that relating multidisciplinary professionalism, organizational expenses, and budgets. George et al. revealed that nurses from a non-shared governance hospital had less engagement in decision making compared to nurses in a shared governance model, which comes in line with our results [15]. However, several studies have reported results supporting nurses' ability to participates in nursing profession committees [12,13].

The findings of this study were consistent with results of a study conducted by Tourangeau et al. [16]; they stated that nurses perceived they have the least extent of control over professional practice; as well as, they perceived slight contribution or control in several areas that affect directly the patients' bedside care from nursing, quality care standards, educational progress, and determining the structure of nursing care for their work. Also, Seada and Etway revealed that nurses had lowermost scores regarding their perception of shared governance which showed that they did not have control over their professional work setting [14].

Shared governance is characterized by collaborative decision-making between management and staff working, together at the organizational level and unit level [17]. The values of shared governance have been united in nursing structures to providing a transformational structure for staff nursing care and enhancing an organization's productivity and performance [18]. The three essential values related to shared governance are the responsibility of delivering for nursing care should belong to clinical staff, nurses' authority for being renowned by the organization, and quality patient care accountability and nursing professionalism must be acknowledged by the clinical staff [6].

Nursing shared governance denotes to the played nurses' role in decision-making and liability for patient care [19]. Shared governance affords the context for a cooperative milieu of nursing leaders and nurses. Both, they can frame a partnership of shared decision for operational and clinical practices [18]. Worldwide, nurses are the prime profession in healthcare settings. Therefore, participation and influence in shared governance are necessary to enable constant transformation, advance and progress of the nursing profession. Bringing this to reality, fitting models, and processes that deliver and inspire participation in decision-making are indispensable [6]. 

Limitations

Limitations of this study include the employment of basic descriptive statistics. A further bivariate and multivariate analysis could be considered in the future. Other acknowledged limitations are the relatively modest sample size and limited study site; therefore, the findings may not be representative of the entire. A prospective study with a bigger sample size and multi-settings will be powerful to generalize the data.

## Conclusions

The results showed that the studied setting lacks a shared governance model in place to engage nurses in decision-making which can enable them to control their professional practice. The findings of our study showed a traditional management governance type in the study setting. The study findings can be utilized to enhance nurses' work milieu and improve shared governance. These results would be an added value for nursing top management to enhance nurses’ perception of shared governance by emerging or implementing shared governance model. In the light of our findings, nurse managers must adopt and apply strategies to empower nurses like shared governance that provide nurses the chance to control their nursing practice and improve quality nursing care.
